# High-Resolution Mapping of Local Photoluminescence Properties in CuO/Cu_2_O Semiconductor Bi-Layers by Using Synchrotron Radiation

**DOI:** 10.3390/ma14195570

**Published:** 2021-09-25

**Authors:** Masakazu Kobayashi, Masanobu Izaki, Pei Loon Khoo, Tsutomu Shinagawa, Akihisa Takeuchi, Kentaro Uesugi

**Affiliations:** 1 Department of Mechanical Engineering, Toyohashi University of Technology, Toyohashi 441-8580, Japan; m-izaki@me.tut.ac.jp (M.I.); khoo@tf.me.tut.ac.jp (P.L.K.); 2 Osaka Research Institute of Industrial Science and Technology, Osaka 536-8553, Japan; tshina@omtri.or.jp; 3 Japan Synchrotron Radiation Research Institute, Sayo 679-5198, Japan; take@spring8.or.jp (A.T.); ueken@spring8.or.jp (K.U.)

**Keywords:** oxide semiconductor, electrodeposition, photoluminescence, focused X-ray, imaging

## Abstract

The quality of a semiconductor, which strongly affects its performance, can be estimated by its photoluminescence, which closely relates to the defect and impurity energy levels. In light of this, it is necessary to have a measurement method for photoluminescence properties with spatial resolution at the sub-micron or nanoscale. In this study, a mapping method for local photoluminescence properties was developed using a focused synchrotron radiation X-ray beam to evaluate localized photoluminescence in bi-layered semiconductors. CuO/Cu_2_O/ZnO semiconductors were prepared on F:SnO_2_/soda-lime glass substrates by means of electrodeposition. The synchrotron radiation experiment was conducted at the beamline 20XU in the Japanese synchrotron radiation facility, SPring-8. By mounting the high-sensitivity spectrum analyzer near the edge of the CuO/Cu_2_O/ZnO devices, luminescence maps of the semiconductor were obtained with unit sizes of 0.3 μm × 0.3 μm. The devices were scanned in 2D. Light emission 2D maps were created by classifying the obtained spectra based on emission energy already reported by M. Izaki, et al. Band-like structures corresponding to the stacking layers of CuO/Cu_2_O/ZnO were visualized. The intensities of emissions at different energies at each position can be associated with localized photovoltaic properties. This result suggests the validity of the method for investigation of localized photoluminescence related to the semiconductor quality.

## 1. Introduction

Multi-layered solar cell devices have been proposed and designed to improve photovoltaics performance (e.g., [1]). For obtaining good photovoltaics performance, it is necessary to understand the influences of layered interface mismatches and boundary segregated impurities on the photovoltaic properties of multi-layered film semiconductor devices (e.g., [2,3]). To investigate local physical properties, such as photoluminescence (PL), at complex heterogeneous interfaces and boundaries, an inspection technique that can be associated with local structure is necessary. However, conventional PL measurements, which ordinarily cover a wide inspection area, are not suitable for the investigation of individual film layers and their interfaces. Frazer et al. reported the relationships between luminescence imaging and lattice defects in Cu_2_O crystals fabricated by the floating zone method [4]. Here, luminescence imaging by the excitation of a laser beam was utilized and mapped onto a region of a few hundred micrometers. However, such resolution is still insufficient to investigate the local PL within a multi-layered film device with the size of several tens of micrometers, although the resolution has been improved year after year [5].

It has been pointed out that photoluminescence and photovoltaic properties are affected by the presence of lattice defects, such as vacancies and impurities in semiconductor materials and devices [4,6]. Moreover, in multi-layered film semiconductor devices that possess complex heterogeneous structures, local variations of photovoltaic properties are expected to accompany heterogeneity and lack of lattice defects. As such, a method to locally characterize photoluminescence and photovoltaic properties should be developed. Research to directly link the local physical properties to the local structures of bulk devices (e.g., vacancies, impurities, crystal boundaries, interfaces and so on) is likely necessary for finding the best solution of layer structure. Additionally, if the investigation can be conducted non-destructively, the study of property changes during use and after a long period of use would also be possible.

A luminescence spectrograph utilizing synchrotron radiation, named SUPERLUMI, was developed at HASYLAB in the 1990s [7,8] and can measure luminescence properties at high precision [9,10]. Here, high-brilliance synchrotron radiation improved time resolution [7,8]. However, spatial resolution was limited and deemed insufficient. Currently, scanning X-ray microscopy at a synchrotron radiation facility is available, with a 65 nm-size focusing beam [11]. We proposed and applied an imaging technique for local photoluminescence mapping by means of a high-intensity focused X-ray at the undulator beam line in the Japanese synchrotron radiation facility, SPring-8, in our previous research [12]. The spatial distribution of localized photoluminescence in a CuO/Cu_2_O semiconductor was measured and demonstrated with a grid size of 0.3 μm × 5 μm.

It is noteworthy that Cu_2_O films have recently gained increased attention in the field of photoactive devices, such as photovoltaics (e.g., [13,14,15]), photonic crystals (e.g., [16]) and photocatalysts (e.g., [17]), due to their optical and electrical characteristics (e.g., [18,19]). The CuO/Cu_2_O bi-layer is a potential candidate material for high-performance photoactive material for solar cells, as well as for photocathodes to generate hydrogen by photoelectrochemical water splitting. The bi-layer includes two p-type semiconductors with different bandgap energies, which is a strategy to realize a high-performance photovoltaic layer by extending the photovoltaic wavelength range and improving the quantum efficiency [6]. Since the photovoltaic performance is highly dependent on its semiconductor quality, which affects carrier transportation and recombination loss [6], the importance of local photoluminescence that allows the investigation of local semiconductor quality is evident.

The Cu_2_O/CuO bi-layered film utilized in the previous research [12] was prepared form the electrodeposited Cu_2_O films by annealing at 673 K for 3.6 ks (1 h) in air [20]. It has been reported that the luminescence properties of the electrodeposited Cu_2_O and CuO formed by annealing are different [21]. Currently, hybrid composites such as the bi-layered film of CuO/Cu_2_O attract the interest of many, and have been investigated as high-efficiency photocathodes for photoelectrochemical hydrogen evolution reaction [22,23] and electrode materials for batteries [24,25]. The Cu_2_O/CuO bi-layered film, the luminescence properties of which were reported and established, is very suitable for sample evaluation in this study to test and develop the improved local mapping method. The FTO film functions as a transparent conductive layer to make electrodeposition possible, while ZnO plays the roles of a conductive film and an n-type semiconductor that forms an n-p junction with a p-type semiconductor of Cu_2_O.

In this study, the further improvement of spatial resolution was attempted by using a fine focused X-ray beam set-up (beamline 20XU, Japan Synchrotron Radiation Research Institute (SPring-8), Sayo, Hyogo, Japan) [11]. Furthermore, the energy detection range was extended by installing a high-sensitivity PL-detector (Otsuka Electronics Co., Ltd., Hirakata-shi, Osaka, Japan). It was noticed that a trade-off relationship between the resolution and detection exists, because a smaller beam makes PL detection difficult. The problems that came to light while conducting this experiment and the possibility of an improved method are discussed.

## 2. Materials and Methods

### 2.1. Samples

The experimental sample for this study is a bi-layer film constituted of cupric oxide (CuO) and cuprous oxide (Cu_2_O). The film was prepared on F:SnO_2_ (FTO)/soda-lime glass (SLG) substrates (AGC Fabritech Co. Ltd., Minato, Tokyo, Japan) by electrodeposition in an aqueous solution [26,27]. First, the ZnO layer was prepared by electrodeposition on the substrate in an aqueous solution containing an 80 mmol/L zinc nitrate hydrate (Nacalai Tesque Inc., Nakagyo, Kyoto, Japan) at −0.8 V referenced to an Ag/AgCl electrode and 335 K for an electric charge of 0.5 C cm^−2^ using a potentiostat (Hokuto Denko, HAL 3000, Megro, Tokyo, Japan) connected to a coulomb meter (Hokuto Denko, HF 301, Megro, Tokyo, Japan). The solution was prepared using reagent grade chemicals and deionized water (purified with Milli Pore Ellix-UV-Advantage) (Merck KGaA, Darmstadt, Germany). Next, an aqueous solution with a pH of 13.0 and containing a 0.3 mol/L copper (II) sulfate hydrate, 0.3 mol/L tartaric acid, and 1.5 mol/L sodium hydroxide was used for the electrodeposition of the CuO/Cu_2_O bi-layer. The CuO/Cu_2_O bi-layer was fabricated by automatically switching the potential at 0.4 V for the CuO layer and at −0.4 V for the Cu_2_O layer for a total absolute electric charge of 1 C cm^−2^ at 323 K with a polarization system (Hokuto Denko, HSV-110, Megro, Tokyo, Japan) under light-irradiation by a high-pressure mercury lamp (USHIO, OPTICAL-MODULEX, 500W) (Ushio, Inc., Chiyoda, Tokyo, Japan) [28]. Ag/AgCl and Pt electrodes were used as the reference and counter electrodes. Subsequently, the samples were cut into smaller specimens with the dimension of 5 mm square using a glass-cutter for the synchrotron experiment.

**Figure 1:**
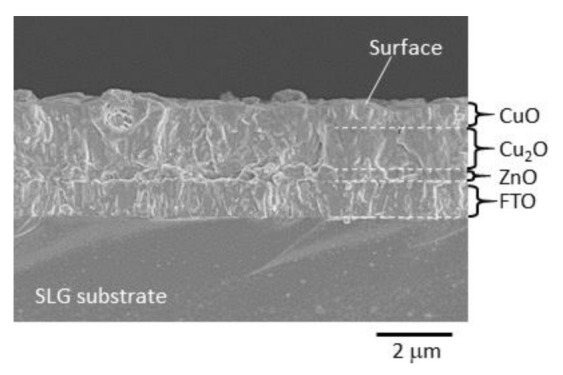
The cross-sectional SEM image for the CuO/Cu_2_O bi-layered film formed on a ZnO/FTO-coated substrate.

Figure 1 shows the SEM (JEOL Ltd., JSM6700F, Akishima, Tokyo, Japan) image of the cross section of the prepared sample. The stacked layers of ZnO, Cu_2_O and CuO can be viewed in this cross-sectional image. The thickness of the upper CuO layer observed here is thinner than the lower Cu_2_O layer. The ZnO layer is observed near the FTO substrate. The thicknesses of ZnO, Cu_2_O, and CuO were approximated at 0.3 μm, 1.3 μm, and 0.8 μm, respectively. The total thickness of the film was approximately 3–4 μm. The structures of the prepared Cu_2_O and CuO films by a similar process have also been confirmed by means of XRD (Rigaku Corp., RINT 2500, Akishima, Tokyo, Japan) inspection, as reported by Izaki et al. [28].

### 2.2. Synchrotron Experiment

**Figure 2:**
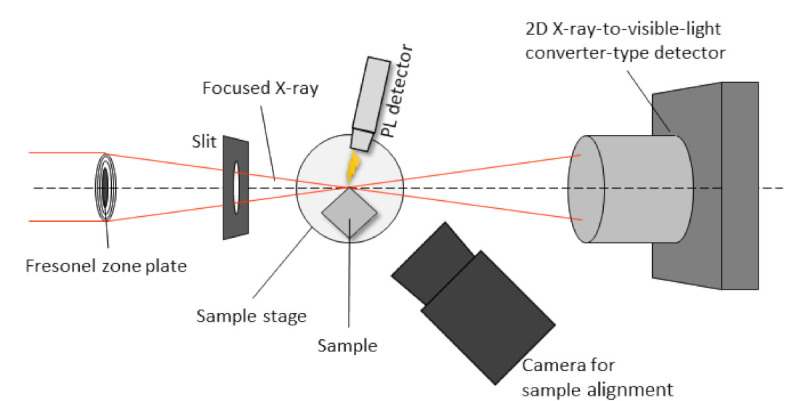
The experimental set-up used in this study.

PL mapping was carried out at the first experimental hutch of BL20XU in the Japanese synchrotron facility, SPring-8. A schematic illustration of the experimental set-up used in this study is shown in Figure 2. The monochromatic X-ray energy of 10 keV was chosen by using an (111) Si double crystal monochromator (standard type of SPring-8, Japan). A probe beam was generated by using a Fresnel zone plate (FZP) as a focal beam [11]. The sample was set at the focal plane of the FZP. The FZP’s zone material was made of tantalum with 1 μm thickness and a diameter of 310 μm, as well as an outermost zone width of 50 nm and a focal length of 625 mm at 10 keV. Vertical and horizontal slits were installed to cut off scattering beams. The size of the focused beam can be estimated at 0.3 μm in width and 0.3 μm in height of the sample position. The width of the beam used in this study was approximately 16 times smaller than the beam used in the previous research [12].

**Figure 3:**
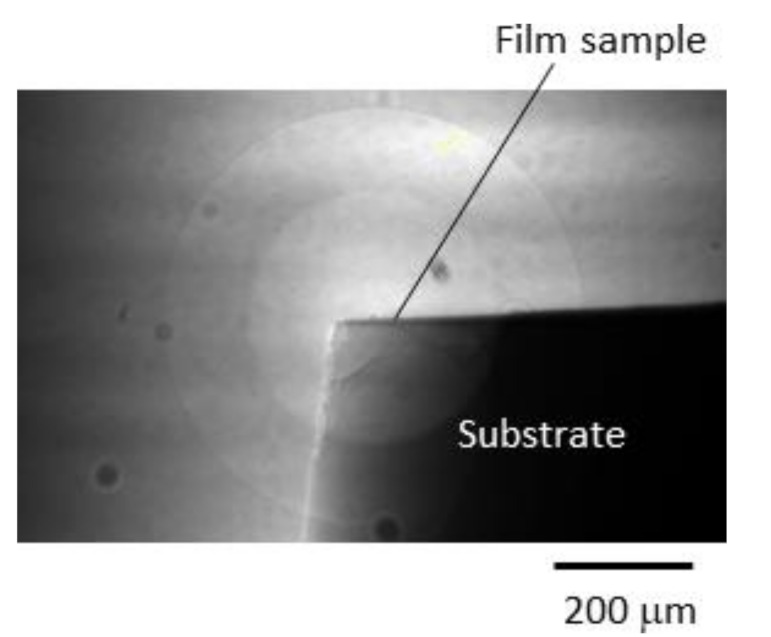
Radiograph obtained by the 2D X-ray detector.

A small piece of the deposited CuO/Cu_2_O/ZnO substrate was mounted horizontally on a stage, and a focused X-ray beam was irradiated at the square corner of the sample, as shown in Figure 2. The sample stage had high-precision drive mechanisms for the horizontal, vertical, and rotational movement. Initially, the sample position was roughly adjusted based on the camera image for sample alignment. The detailed position was further calibrated using a 2D X-ray-to-visible light converter-type detector, which consisted of a scintillator, an optical lens, and a CMOS camera (Hamamatsu Photonics K.K., ORCA-Flash4.0, Hamamatsu, Shizuoka, Japan), which was placed 150 mm behind the sample (see Figure 3).

A light-receiving fiber for the PL emission and an array spectrometer MCPD-9800 (Otsuka Electronics Co., Ltd., Hirakata-shi, Osaka, Japan) were placed roughly perpendicular to the X-ray beam. The spectrometer consisted of flexible optical fiber, slits, grating, and array-detecting elements. It can measure light with wavelengths of 360–1100 nm with high sensitivity. The light-receiving fiber was installed at the position where the intensity of light emission became the maximum. The X-ray beam intensity was sufficient to observe PL emissions in the sample of this study. Two-dimensional scans of the PL (i.e., 2D mapping) were performed on the CuO/Cu_2_O bi-layered film. The emission spectra were collected by the spectrometer for an exposure time of 10 s at each X-ray irradiation position.

## 3. Results and Discussion

**Figure 4:**
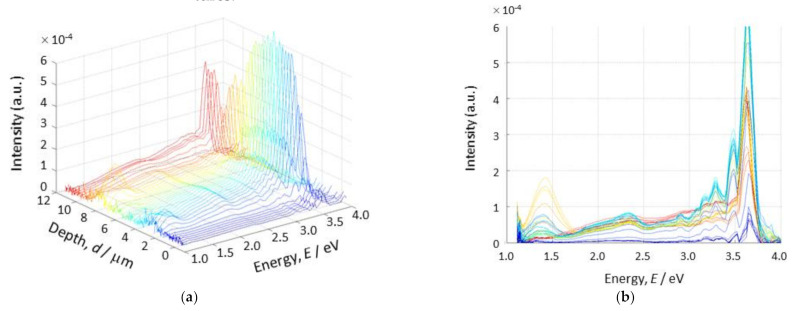
The photoluminescence spectra obtained by the spectrometer during a depth scan. (**a**) Three-axis plot of the emission energy, position (depth), and emission intensity, and (**b**) intensity vs. energy, in which several peaks can be found.

The photoluminescence spectra obtained by the spectrometer are shown in Figure 4. In this case, the focused synchrotron radiation beam scanned the CuO/Cu_2_O film in the depth direction by steps of 0.3 μm. Peaks around 1.4 eV, 2.4 eV, and 3.3 eV were found in the spectra, depending on the scanned depth. Very strong peaks around the vicinity of 3.6 eV are found in almost all of the scanned positions.

According to the reported references [20,26,29], 2.0 eV–2.1 eV visible light emitted by Cu_2_O can be attributed to the direct recombination of the photon-assisted excitons. Additionally, Cu_2_O emitted 1.52 eV-light as a defect-related emission [26,30,31]). With regard to CuO, it was reported that the light emission at 1.32 eV [32], 1.4 eV [33], and 1.38–1.56 eV [34] were due to bandgap energies. It was also reported that the (0001)-oriented ZnO layer emits not only near-band emissions at 3.25 eV –3.3 eV by recombination [35], but also visible light emissions at 2.28 eV and 2.8 eV [36]. In addition, the substrates possess emission peaks at 1.9 eV and 2.7 eV [12]. The obtained peaks in Figure 4 are related to the emission light energies reported for each material constituting the film sample. It was noted that the intensity of light emission was weak compared to the previous work [12], due to a smaller-sized X-ray beam. Nonetheless, despite reducing the X-ray beam’s size, the local measurement of photoluminescence spectra was successful, although a delicate set-up for the spectrometer was necessary. The light emission spectra obtained at each position were then converted into intensity maps with individual energies, in order to understand the detailed relationship between localized luminescence and film structures.

**Figure 5:**
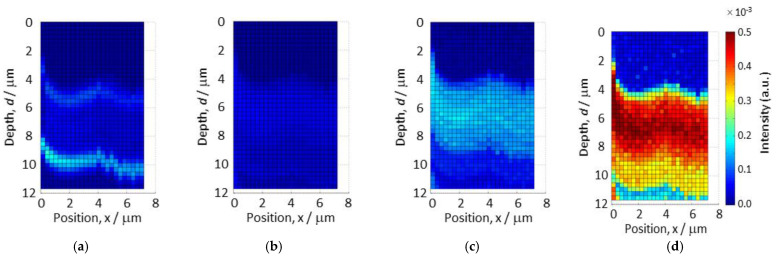
Light emission maps at different energies: (**a**) 1.4 eV, (**b**) 2.0 eV, (**c**) 3.3 eV, and (**d**) 3.6 eV, in CuO/Cu_2_O bi-layered film prepared on the ZnO/FTO/SLG substrate.

Figure 5 shows the intensity maps of the measured emissions at (a) 1.4 eV, (b) 2.0 eV, (c) 3.3 eV, and (d) 3.6 eV. The vertical and horizontal axes of the figure correspond to the directions parallel to the film’s depth and the substrate’s surface plane, respectively. In the absence of light emission, we found the film surface placed at the depth position of about 4 μm. Horizontal bands of high intensities are clearly visible in (a), (c), and (d). An extremely weak emission was observed for the whole area of the sample in (b). The high-intensity bands observed in (a), (c), and (d) are not flat. This seems to reflect the film structures at an improved spatial resolution by using the finer focus beam. However, the initial curves of intensity bands observed in the vicinity of zero-position of the horizontal x-axis may have been due to the initial X-ray beam drift.

The emissions observed at 1.4 eV can be associated with Cu_2_O and CuO, as mentioned earlier in this section. Two separated narrow bands are recognized in (a), although CuO and Cu_2_O layers are stacked in the prepared film. The intensity of the band nearer to the surface is weaker than that of the lower band. Emissions of 2.0 eV shown in (b) are expected in Cu_2_O. However, no clear light emission was obtained in this study; only weak light was observed. Light emissions at 3.3 eV could not be obtained in the previous work [12] because 3.3 eV was out of range for the spectrometer used in the last experiment. The emission at 3.3 eV can be related to the ZnO layer. As shown in the map (c), light emissions were obtained in a depth range from 4 μm to 8 μm. In this study, it was found that not only spatial resolution but also energy range was extended. When one observes the 3.6 eV map (d), which would almost reflect the whole sample structure in detail, it is revealed that the film thickness observed from the light emission map of the X-ray beam is thicker than that observed by SEM (see Figure 1). The SEM observation of the samples was carried out again after the synchrotron experiment, to confirm the experimental situation of the X-ray scan.

**Figure 6:**
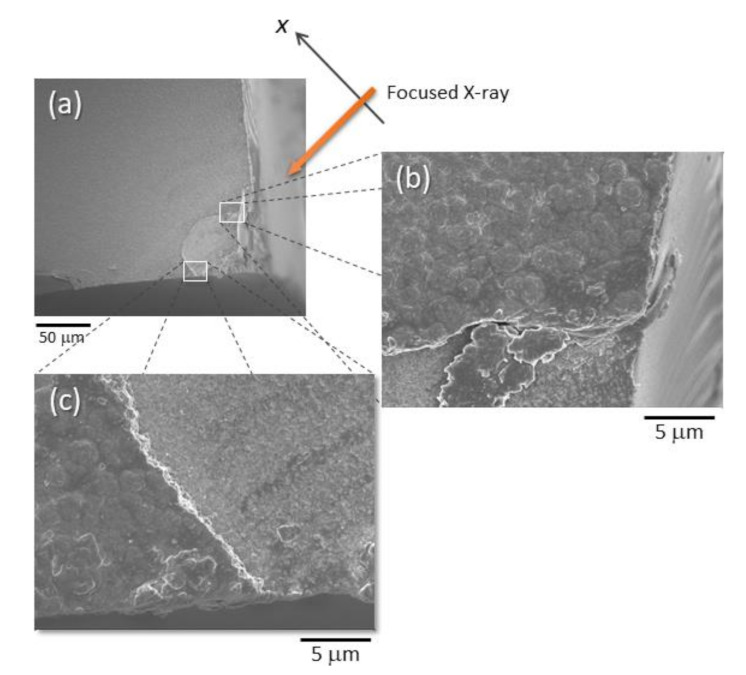
Top views of X-ray scanned sample observed by SEM. Image of the sample corner, which was scanned by X-ray (indicated by an arrow), in low magnification are shown in (**a**). The magnified images within (**a**) are shown in (**b**,**c**).

**Figure 7:**
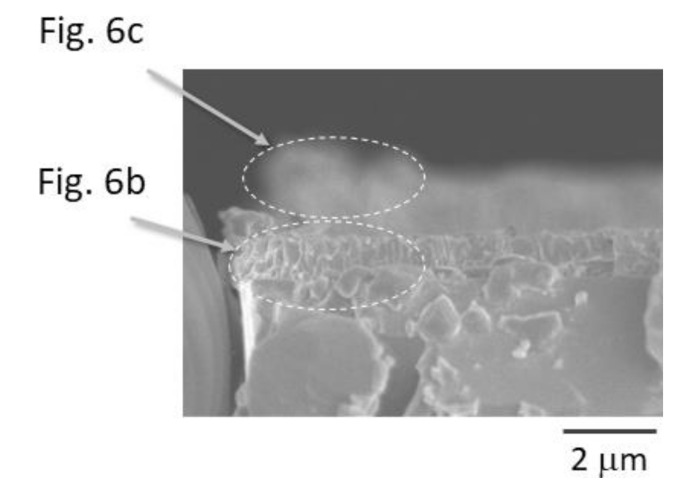
Side view of the X-ray-scanned sample by SEM.

Figure 6 and Figure 7 show the SEM images of the X-ray scanned sample. Top views are shown in Figure 6. A missing part of the film is recognized at the corner of the substrate, as shown in (a). This was due to the pushing and cracking of the substrates with a glass cutter while the small samples were being prepared to be mounted onto the stage. The shape of the missing area was arc-shaped. Magnified images at the end of the arc can be seen in (b) and (c). The bi-layered CuO/Cu_2_O film seemed to be undamaged by the X-ray irradiation. The non-flat surface in the targeted area observed in the SEM images corresponds to the surface characteristic obtained in Figure 5.

The sample image observed from the side of X-ray irradiation is shown in Figure 7. The two arc edges are shown in Figure 6b,c. Since the mounting of the substrate on sample stages was reproducible for both the X-ray experiment and SEM observation, this shows that there was a probable difference in film height at different film positions. The difference of film thickness in the X-ray scan (Figure 5) and SEM image (Figure 1) can also be explained. Two narrow bands were observed at 1.4 eV separately, as shown in Figure 5a. These emissions came from the CuO and Cu_2_O layers. The origin of the two observed bands is understandable by referring to the SEM image shown in Figure 7; that is, emissions occurred not only for the front side but also the rear side exposed to the front view. As the light emission was obtained at 3.3 eV, which corresponds to ZnO emissions distributed through a depth range from 4 μm to 8 μm, the mounted substrate might be slightly tilted. The results obtained from the comparison of X-ray scans and SEM observations strongly point to the importance of sample alignment in order to improve the spatial resolution of X-ray scanning. The preparation method of the sample pieces should also be improved. Two-dimensional PL mapping was achieved in this study, but the utilization of a tomographic technique can be considered to obtain a three-dimensional PL map. The tomographic method, which reconstructs cross-sections of the sample, could solve the issues of sample condition and sample alignment, as revealed in this study.

## 4. Conclusions

In this study, the scanning of local photoluminescence was attempted on a CuO/Cu_2_O bi-layered film formed on a ZnO/FTO substrate by a size-reduced, focused X-ray beam in comparison to the previous study. Although there is a trade-off, with the increase in spectrum collection time due to the decrease in beam intensity, the mapping of photoluminescence influenced by microstructures was possible by utilizing a high-sensitivity spectrometer. Acquisition of the two-dimensional PL maps was successfully accomplished through 0.3 × 0.3 step scanning by applying a similar set-up, which recorded a 65 nm-size focusing beam, as reported in [11], although such fine step had only been available for vertical scan in the previous study [12]. The wavelength range measurable by localized photoluminescence was also extended to over 3.1 eV by the spectrometer. However, it was revealed that the utilization of a small beam makes sample alignment difficult. Comparison of PL maps and SEM images of the scanned sample indicated the importance of sample alignment to measure localized PL correctly. Proper care should also be taken to prevent the film from being damaged. These results gave insight for future trials and improvisations towards a high-resolution localized PL mapping, which are expected in the near future.

## Data Availability

Data will be made available upon reasonable request.

## References

[1] Lee T.D., Ebong A.U. (2017). A review of thin film solar cell technologies and challenges. Renew Sustain. Energy Rev..

[2] Musselman K.P., Marin A., Schmidt-Mende L., MacManus-Driscoll J.L. (2012). Incompatible Length Scales in Nanostructured Cu_2_O Solar Cells. Adv. Fanct. Mater..

[3] Wang Y., Steigert A., Yin G., Parvan V., Klenk R., Schlatmann R., Lauermann I. (2017). Cu_2_O as a Potential Intermediate Transparent Conducting Oxide Layer for Monolithic Perovskite-CIGSe Tandem Solar Cells. Phys. State Solidi C.

[4] Frazer L., Lenferink E.J., Chang K.B., Poeppelmeier K.R., Stern N.P., Ketterson J.B. (2015). Evaluation of defects in cuprous oxide through exciton luminescence imaging. J. Lumin..

[5] Rodenbücher C., Gensch T., Speier W., Breuer U., Pilch M., Hardtdegen H., Mikulics M., Zych E., Waser R., Szot K. (2013). Inhomogeneity of donor doping in SrTiO_3_ substrates studied by fluorescence-lifetime imaging microscopy. Appl. Phys. Lett..

[6] Izaki M., Fukazawa K., Sato K., Khoo P.L., Kobayashi M., Takeuchi A., Uesugi K. (2019). Defect Structure and Photovoltaric Characteristics of Internally Stacked CuO/Cu_2_O Photoactive Layer Prepared by Electrodeposition and Heating. ACS Appl. Energy Mater..

[7] Zimmerer G. (2006). Luminescence spectroscopy with synchrotron radiation: History, highlights, future. J. Lumin..

[8] Zimmerer G. (2007). SUPERLUMI: A unique setup for luminescence spectroscopy with synchrotron radiation. Radiat. Meas..

[9] Pankratov V., Popov A.I., Kotlov A., Feldmann C. (2011). Luminescence of nano-and macrosized LaPO_4_:Ce,Tb excited by synchrotron radiation. Opt. Mater..

[10] Zorenko T., Grbenko V., Safronova N., Matveevskaya N., Yavetskiy R., Babayevska N., Zorenko Y. (2018). Comparative study of the luminescent properties of oxide compounds under synchrotron radiation excitation: Lu_2_O_3_:Eu nanopowders, ceramics and films. J. Lumin..

[11] Takeuchi A., Uesugi K., Suzuki Y., Itabashi S., Oda M. (2017). Fresnel zone plate with apodized aperture for hard X-ray Gaussian beam optics. J. Synchrotron Rad..

[12] Kobayashi M., Izaki M., Shinagawa T., Takeuchi A., Uesugi K. (2018). Localized Photoluminescence Imaging of Bi-Layered Cuprous/Cupric Oxide Semiconductor Films by Synchrotron Radiation. Phys. Status Solidi B.

[13] Izaki M., Shinagawa T., Mizuno K.-T., Ida Y., Inaba M., Tasaka A. (2007). Electrochemically constructed p-Cu_2_O/n-ZnO heterojunction diode for photovoltaic device. J. Phys. D Appl. Phys..

[14] Musselman K.P., Wisnet A., Iza D.C., Hasse H.C., Scheu C., MacManus-Discoll J.L., Schmidt-Mende L. (2010). Strong Efficiency Improvements in Ultra-low-Cost Inorganic Nanowire Solar Cells. Adv. Mater..

[15] Zuo C., Ding L. (2015). Solution-Processed Cu_2_O and CuO as Hole Transport Materials for Efficient Perovskite Solar Cells. Small.

[16] Park S.-G., Miyake M., Yang S.-M., Braun P.V., Wiltzius P. (2011). Cu_2_O Inverse Woodpile Photonic Crystals by Prism Holographic Lithography and Electrodeposition. Adv. Mater..

[17] Geng Z., Zhang Y., Yuan X., Huo M., Zhao Y., Lu Y., Qiu Y. (2015). Incorporation of Cu_2_O nanocrystals into TiO_2_ photonic crystal for enhanced UV–visible light driven photocatalysis. J. Alloy. Comp..

[18] Grez P., Herrera F., Riveros G., Ramírez A., Henríquez R., Dalchiele E., Schrebler R. (2012). Morphological, structural, and photoelectrochemical characterization of n-type Cu_2_O thin films obtained by electrodeposition. Phys. Status Solidi A.

[19] Benz J., Hering K.P., Kramm B., Polity A., Klar P.J., Siah S.C., Buonassis T. (2017). The influence of nitrogen doping on the electrical and vibrational properties of Cu_2_O. Phys. Status Solidi B.

[20] Meyer B.K., Polity A., Rappin D., Becker M., Hering P., Klar P.J., Sander T., Reindl C., Benz J., Eickhoff M. (2012). Binary copper oxide semiconductors: From materials towards devices. Phys. Status Solidi B.

[21] Chang K.B., Frazer L., Schwartz J.J., Ketterson J.B., Poeppelmeier K.R. (2013). Removal of Copper Vacancies in Cuprous Oxide Single Crystals Grown by the Floating Zone Method. Cryst. Growth Des..

[22] Yang Y., Xu D., Wu Q., Diao P. (2016). Cu_2_O/CuO Bilayered Composite as a High-Efficiency Photocathode for Photoelectrochemical Hydrogen Evolution Reaction. Sci. Rep..

[23] Jamali S., Moshaii A., Mohammadian N. (2017). Improvement of Photoelectrochemical and Stability Properties of Electrodeposited Cu_2_O Thin Films by Annealing Processes. Phys. Status Solidi A.

[24] Kim A.-Y., Kim M.K., Cho K., Woo J.-Y., Lee Y., Han S.-H., Byun D., Choi W., Lee J.K. (2016). One-Step Catalytic Synthesis of CuO/Cu_2_O in a Graphitized Porous C Matrix Derived from the Cu-Based Metal-Organic Framework for Li- and Na-Ion Batteries. ACS Appl. Mater. Interfaces.

[25] Wu S., Fu G., Lv W., Wei J., Chen W., Yi H., Gu M., Bai X., Zhu L., Tan C. (2018). A Single-Step Hydrothermal Route to 3D Hierarchical Cu_2_O/CuO/rGO Nanosheets as High-Performance Anode of Lithium-Ion Batteries. Small.

[26] Izaki M., Sasaki S., Mohamad F.B., Shinagawa T., Ohta T., Watase S., Sasano J. (2012). Effects of preparation temperature on optical and electrical characteristics of (111)-oriented Cu_2_O films electrodeposited on (111)-Au film. Thin Solid Film..

[27] Shinagawa T., Onoda M., Fariza B.M., Sasano J., Izaki M. (2013). Annealing effects and photoelectric properties of single-oriented Cu_2_O films electrodeposited on Au(111)/Si(100) substrates. J. Mater. Chem. A.

[28] Izaki M., Koyama T., Khoo P.L., Shinagawa T. (2020). Light-Irradiated Electrochemical Direct Construction of Cu_2_O/CuO Bilayers by Switching Cathodic/Anodic Polarization in Copper(II)-Tartrate Complex Aqueous Solution. ACS Omega.

[29] Ray S.C. (2001). Preparation of copper oxide thin film by the sol-gel-like dip technique and study of their structural and optical properties. Sol. Energy Mater. Sol. Cells.

[30] Terui Y., Fujita M., Miyakita Y., Sogoshi N., Nakabayashi S. (2005). Photoluminescence of Electrochemically-Deposited Granular Cu_2_O Films. Trans. Mater. Res. Soc. Jpn..

[31] Scanlon D.O., Morgan B.J., Watson G.W. (2009). Modeling the polaronic nature of p-type defects in Cu_2_O: The failure of GGA and GGA+U. J. Chem. Phys..

[32] Wang L., Han K., Tao M. (2007). Effect of Substrate Etching on Electrical Properties of Electrochemically Deposited CuO. J. Electrochem. Soc..

[33] Izaki M., Nagai M., Maeda K., Farina F.B., Motomura K., Sasano J., Shinagawa T., Watase S. (2011). Electrodeposition of 1.4-eV-Bandgap p-Copper (II) Oxide Film with Excellent Photoactivity. J. Electrochem. Soc..

[34] Nakaoka K., Ueyama J., Ogura K. (2004). Photoelectrochemical Behavior of Electrodeposited CuO and Cu_2_O Thin Films on Conducting Substrates. J. Electrochem. Soc..

[35] Yamamoto A., Miyajima K., Goto T., Ko H.J., Yao T. (2001). Biexciton luminescence in high-quality ZnO epitaxial thin films. J. Appl. Phys..

[36] Izaki M., Watase S., Takahashi H. (2003). Low-Temperature Electrodeposition of Room-Temperature Ultraviolet-Light-Emitting Zinc Oxide. Adv. Mater..

